# Prospective Antiviral Effect of *Ulva lactuca* Aqueous Extract against COVID-19 Infection

**DOI:** 10.3390/md22010030

**Published:** 2023-12-30

**Authors:** Reem Binsuwaidan, Thanaa A. El-Masry, Mostafa El-Sheekh, Mohamed G. Seadawy, Mofida E. M. Makhlof, Shaimaa M. Aboukhatwa, Nagla A. El-Shitany, Kadreya E. Elmorshedy, Maysa M. F. El-Nagar, Maisra M. El-Bouseary

**Affiliations:** 1 Department of Pharmaceutical Sciences, College of Pharmacy, Princess Nourah bint Abdulrahman University, P.O. Box 84428, Riyadh 11671, Saudi Arabia; rabinsuwaidan@pnu.edu.sa; 2 Department of Pharmacology and Toxicology, Faculty of Pharmacy, Tanta University, Tanta 31527, Egypt; naglaa.fouad@pharm.tanta.edu.eg; 3 Botany Department, Faculty of Science, Tanta University, Tanta 31527, Egypt; mostafaelsheikh@science.tanta.edu.eg; 4 Biological Prevention Department, Egypt Army, Cairo 31527, Egypt; biologist202054@yahoo.com; 5 Botany and Microbiology Department, Faculty of Science, Damanhour University, Damanhour 22511, Egypt; mofida_makhlof@yahoo.com; 6 Department of Pharmaceutical Chemistry, Faculty of Pharmacy, Tanta University, Tanta 31527, Egypt; shaymaa.aboukhatwa@pharm.tanta.edu.eg; 7 Department of Anatomy, Faculty of Medicine, Tanta University, Tanta 31527, Egypt; kadreyaelmorshedy@med.tanta.edu.eg; 8 Department of Microbiology and Immunology, Faculty of Pharmacy, Tanta University, Tanta 31527, Egypt; maysra_mohamed@pharm.tanta.edu.eg

**Keywords:** antiviral, anti-SARS-CoV-2, Egypt, ulvan, *Ulva lactuca*

## Abstract

Marine algal extracts exhibit a potent inhibitory effect against several enveloped and non-enveloped viruses. The infection of severe acute respiratory syndrome coronavirus 2 (SARS-CoV-2) has several adverse effects, including an increased mortality rate. The anti-COVID-19 agents are still limited; this issue requires exploring novel, effective anti-SARS-CoV-2 therapeutic approaches. This study investigated the antiviral activity of an aqueous extract of *Ulva lactuca*, which was collected from the Gulf of Suez, Egypt. The aqueous extract of *Ulva lactuca* was characterized by high-performance liquid chromatography (HPLC), Fourier-transform infrared spectroscopy (FTIR), X-ray diffraction (XRD), and Energy Dispersive X-ray (EDX) analyses. According to the HPLC analysis, the extract comprises several sugars, mostly rhamnose (32.88%). The FTIR spectra showed numerous bands related to the functional groups. EDX analysis confirmed the presence of different elements, such as oxygen (O), carbon (C), sulfur (S), magnesium (Mg), potassium (K), calcium (Ca), and sodium (Na), with different concentrations. The aqueous extract of *U. lactuca* (0.0312 mg/mL) exhibited potent anti-SARS-CoV-2 activity via virucidal activity, inhibition of viral replication, and interference with viral adsorption (% inhibitions of 64%, 33.3%, and 31.1%, respectively). Consequently, ulvan could be a promising compound for preclinical study in the drug development process to combat SARS-CoV-2.

## 1. Introduction

Many scientists believe that studying macroalgae offers a great chance to unearth an endless supply of novel bioactive substances that can be employed as medicines [1]. These aquatic creatures can produce various metabolites, such as polysaccharides, vitamins, amino acids, and halogenated substances [1,2,3]. Recent research has demonstrated that a wide range of active ingredients identified in algae can combat cancer, viruses, and bacteria; reduce inflammatory responses; and halt angiogenesis [3,4,5]. The properties of algae polysaccharides differ from those of their naturally existing equivalents in that they are abundant in sulfated and uronic acid residues [6,7,8]. Ulvan primarily comprises a series of monosaccharides: uronic acid, iduronic acid, rhamnose sulfate, or xylose [9]. Sulfated polysaccharides have been shown to have antiviral, anti-inflammatory, antioxidant, anti-arteriosclerosis, and anti-cancer effects [8,9].

Moreover, the algal polysaccharides are beneficial due to their low toxicity, strong biocompatibility, and immunoregulatory properties [6]. Marine-sulfated polysaccharides may be viable options to combat and treat the SARS-CoV-2 infection. This study investigated the interactions between ulvan and RNA-dependent RNA polymerase (RdRp) and spiked proteins from severe acute respiratory syndrome coronavirus 2 (SARS-CoV-2) by docking simulation using MOE 2020.

Viral infection has recently increased in importance as a concern for human health and is one of the main causes of global death. The COVID-19 viral pneumonia was caused by SARS-CoV-2 which first emerged at the end of 2019 [8]. The virus is a close relative of the SARS-CoV virus that initiated the outbreak of atypical pneumonia in 2002–2003. It belongs to *betacoronaviruses* and primarily infects the lungs and digestive system [10,11]. According to previous reports, viral entry to the cell is achieved via membrane fusion or endocytosis after attaching to the angiotensin-converting enzyme 2 (ACE2) cell receptor via spike protein [12,13]. It makes sense to interfere with the S-protein–ACE2 interaction to limit viral transcription and replication and prevent SARS-CoV-2 infection because binding via the spike protein is required for viral entry into the cell [14].

In addition to SARS-CoV-2’s rapid spread and lethality, COVID-19 seriously disrupts global social and economic activity. Furthermore, many COVID-19-rescued patients experienced significant post-illness long-term health problems [15,16,17].

Given that SARS-CoV-2 is an RNA-enclosed virus with plus sense, it becomes pathogenic by fusing with an infected cell’s cytoplasmic membrane. Anti-SARS-CoV-2 medications target three different targets: RNA-dependent RNA polymerase (RdRp), spike (S) protein, and major viral protease (MPro) [18]. The nucleoside analogues favipiravir, ribavirin, remdesivir, and molnupiravir were initially designed to treat other viruses, but they were subsequently repurposed for COVID-19 treatment. Since ribavirin has a poor safety profile and little efficacy, it is not currently used to treat COVID-19. Favipiravir had no advantageous impact on the length of time it required for viral clearance, the requirement for oxygen therapy, or ICU hospitalization [19]. Molnupiravir has the potential to expedite COVID-19 patients’ recovery; nonetheless, it does not yield a noteworthy decrease in fatality and hospitalization rates [18]. According to the available clinical data, lopinavir/ritonavir is not effective in treating COVID-19 patients; however, it may be helpful when combined with complementary therapies (ribavirin, arbidol, and interferons). Additionally, nirmatrelvir/ritonavir shows promising therapeutic results [18].

Since new vaccines have been administered since 2020, several studies [11,17,20] found that mutant strains could jeopardize vaccine-based immunity and protection gained from prior infections with the first strains. While nirmatrelvir/ritonavir, remdesivir, and molnupiravir have been approved by the WHO as antiviral medications for COVID-19, exploring for new antiviral alternatives is still critical to combating SARS-CoV-2. However, discovering and developing antiviral drugs is expensive and time-consuming [21]. Consequently, we must discover our environment and look for natural alternatives with unique compounds that may help us eliminate this epidemic. The present study investigated the potential antiviral effect of ulvan against SARS-CoV-2 to provide novel alternatives to combat COVID-19.

## 2. Results

### 2.1. Algal Collection and Ulvan Extraction

**Figure 1:**
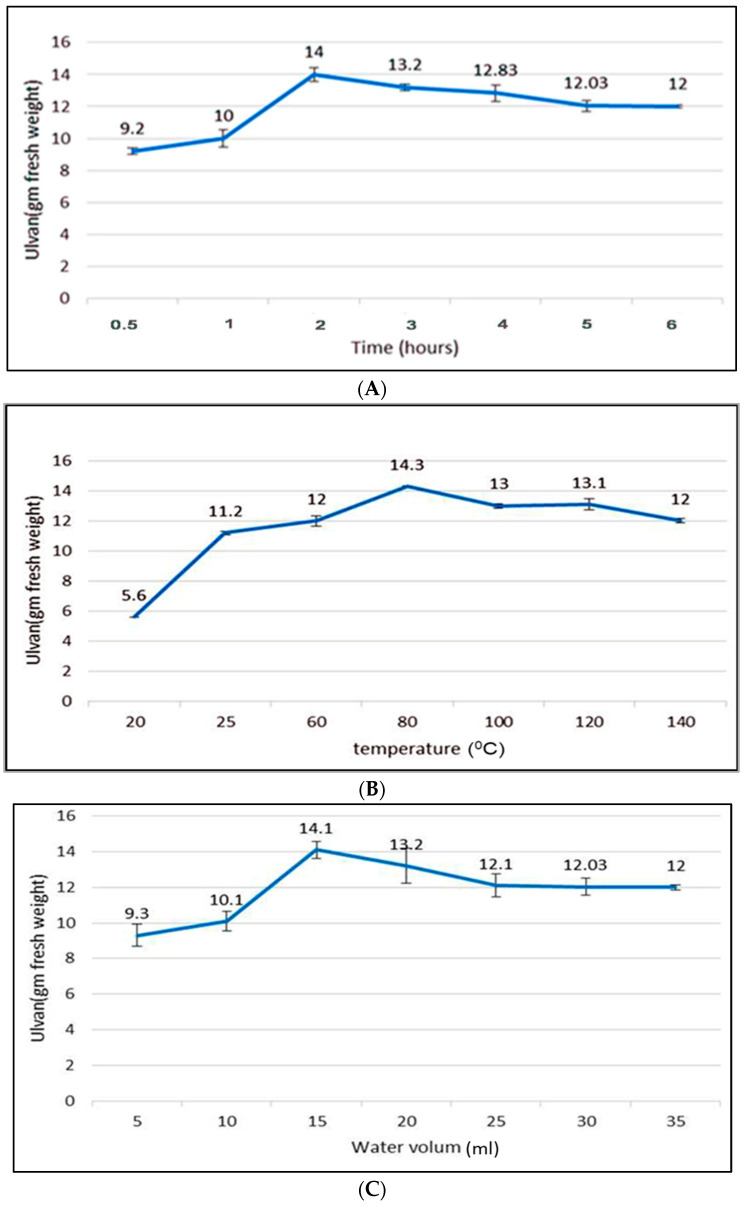
The effect of different extraction conditions on the collected extract of *Ulva lactuca*. (**A**) Periods, (**B**) Temperatures, and (**C**) Water volumes for each 1 g of algal powder.

*Ulva lactuca* was collected in July 2020 from the coast of the Gulf of Suez, Egypt. The aqueous extraction was performed using the hot water extraction method. This study examined key variables, including extraction and temperature, in addition to the impact of varying concentration ratios on the extraction yield (Figure 1). The extraction conditions for the highest extraction yield (~11.203%) were 80 °C, with a 1/20 algal powder/water ratio (g/mL) for 2 h.

### 2.2. Ulvan Characterization

**Figure 2:**
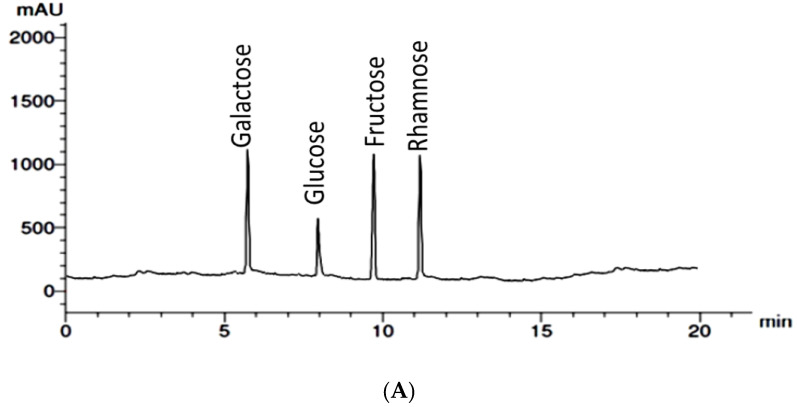

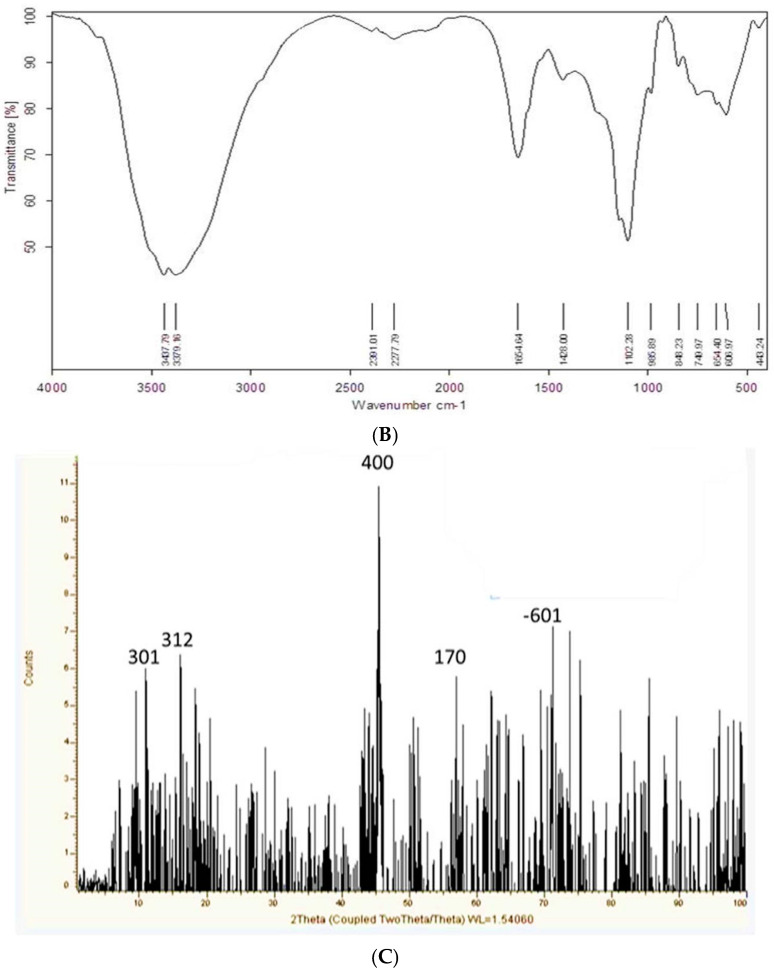

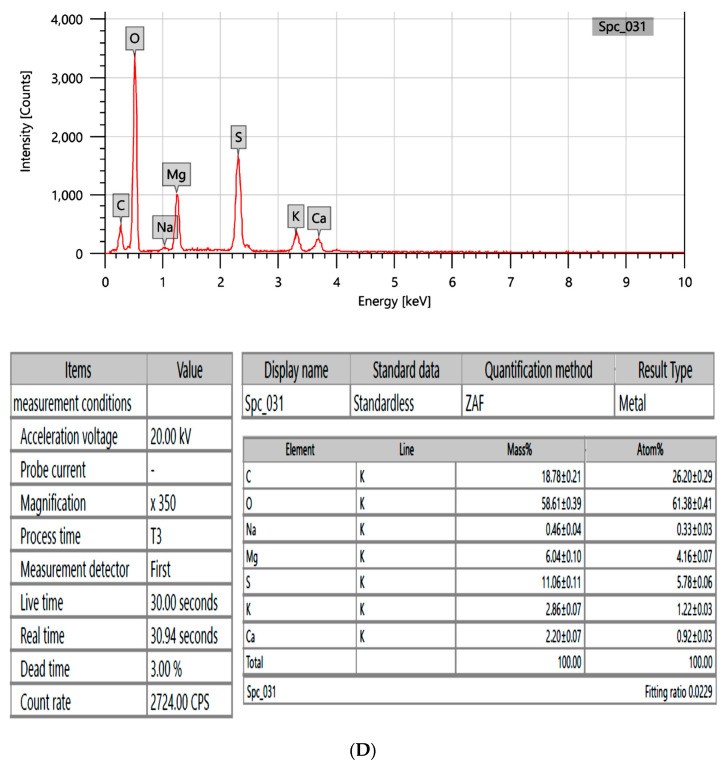
*Ulva lactuca* aqueous extract characterization; (**A**) HPLC Chromatograph; (**B**) infrared spectra between 400 and 4000 cm^−1^; (**C**) the XRD pattern; and (**D**) EDX data.

Table 1 presents the protein and sulfate content of the extract of *U. lactuca* (7.386 and 14.95, respectively). The proximate chemical analysis of *U. lactuca* indicates that our extract contains C, H, N, and S in the percentages of 28.54, 3.61, 2.04, and 8.97, respectively. Figure 2 shows the high-performance liquid chromatography (HPLC) analysis, which revealed that the extract contains extreme amounts of ash (44.004%). Sugars occur at a rate of 33.66%, higher than that recorded by [22], where sugars’ percent (20.09–29.12%).

#### 2.2.1. HPLC Analysis

The aqueous extract of *U. lactuca* was analyzed via HPLC to determine its monosaccharide composition after acid hydrolysis (Table 1) (Figure 2A). HPLC analysis reveals that the *U. lactuca* extract comprises galactose, fructose, rhamnose, and glucose, with rhamnose representing the majority (32.88%). Table 1 presents the dry weight percentage of each sugar monomer in the *U. lactuca* extract. The total rates of sugars resulting from HPLC are less than those observed in total sugar analysis; this indicates that more sugar monomers are not detected by HPLC, such as iduronic acid.

#### 2.2.2. Fourier-Transform Infrared Spectroscopy (FTIR)

The FT-IR spectra revealed numerous bands corresponding to the functional groups of the *U. lactuca* extract (Figure 2B). The absorption band was observed at 3437.7863 and 3379.1576 cm^−1^, corresponding to the polymeric (OH) stretching vibration, which is characteristic of the *U. lactuca* extract. Another band at 2391.0051 and 2277.7889 cm^−1^ is designated for (CH) stretching vibrations, while the band at 1654.6443 and 1427.9982 cm^−1^ is designated for (COO-) stretching vibrations. The sulfated nature of the *U. lactuca* extract was confirmed by the band in the region at 1102.2837 cm^−1^, which is associated with the stretching vibration of the sulfate ester (S=O) group and by the shoulder at 848.2335 cm^−1^. The characteristic of uronic acid residues was also observed in the band at 848.2335 cm^−1^. In contrast, bands of carboxylate groups of uronic acid with comparable intensities are observed in both spectra at around 1654.6443 and 1427.982 cm^−1^. The peaks observed between 749.9708 and 443.2442 cm^−1^ may represent the sugar cycles and have been reported as the typical signature of the *Ulva lactuca* because it is known as the Ulvan fingerprint region.

#### 2.2.3. X-ray Diffraction (XRD)

On collected ulvan samples, XRD patterns were created (Figure 2C). The X-ray diffractograms showed sharp peaks at 2θ = 11°, 17°, 45°, 57°, and 72°, which indicated that ulvan is a polymer having a semi-crystalline structure with a major crystalline reflection at 2θ of 45°.

#### 2.2.4. Energy-Dispersive X-ray (EDX)

The EDX analysis confirmed the presence of different elements, predominantly oxygen (O), carbon (C), sulfur (S), magnesium (Mg), potassium (K), calcium (Ca), and sodium (Na) at 58.61%, 18.78%, 11.06%, 6.04%, 2.86%, 2.20%, and 0.46%, respectively (Figure 2D).

#### 2.2.5. SEM

**Figure 3:**
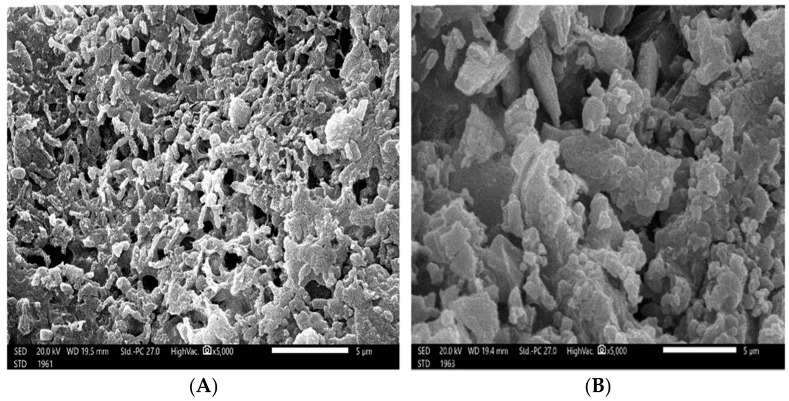
Scanning electron microscopy (SEM) micrographs of the extracted ulvan with different fields showing different crystals’ shapes (**A**,**B**).

The SEM micrographs of the extracted ulvan verified a semi-crystalline and non-smooth texture (Figure 3).

### 2.3. Antiviral Activity of Ulva lactuca

#### 2.3.1. Cytotoxicity Assay

**Figure 4:**
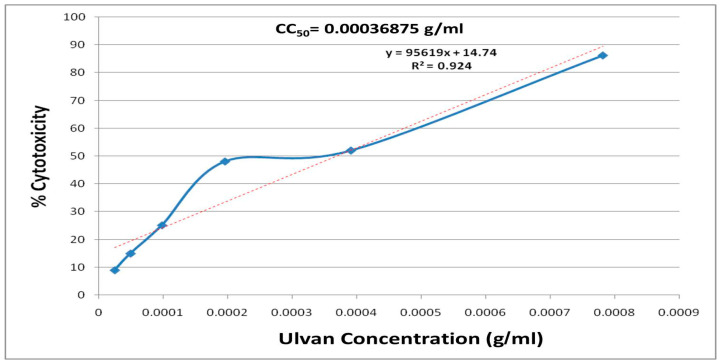
The cytotoxicity of *U. lactuca* aqueous extract on Vero-E6 cells using MTT assay.

The CC_50_ of ulvan was determined using the MTT assay on the Vero-E6 cells to identify the proper *U. lactuca* concentration for investigating its anti-SARS-CoV-2 activity. The CC_50_ of ulvan equals 0.36875 mg/mL (Figure 4).

#### 2.3.2. Plaque Reduction Assay

**Figure 5:**
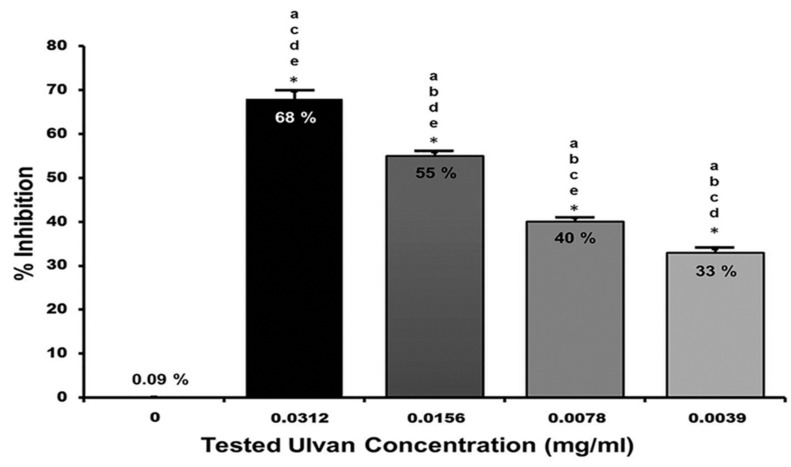
Plaque Reduction Assay results. The experiment was performed in three independent replicates. Data were represented as mean ± SD. ANOVA test followed by a Tukey–Kramer post hoc test, which showed a significant difference among means. The asterisk indicated significant differences of *p* < 0.001. The letters above the bars denote the significant difference across the investigated groups: a = 0 mg/mL, b = 0.0312 mg/mL, c = 0.0156 mg/mL, d = 0.0078 mg/mL, and e = 0.0039 mg/mL.

Figure 5 presents the percentage of inhibition compared to untreated cell cultures. The percentages of inhibition of the tested aqueous extract of *U. lactuca* at concentrations of 0.0312, 0.0156, 0.0078, and 0.0039 mg/mL were 68, 55, 40, and 33%, respectively. An ANOVA test followed by a Tukey–Kramer post hoc test was performed to compare the different investigated groups, which showed a significant difference among means (*p* ˂ 0.001). The IC_50_ of the tested aqueous extract of *U. lactuca* was 0.0154 mg/mL, with a selectivity index (SI) of 23.94.

#### 2.3.3. Mechanism of Antiviral Action

The percentage inhibition of various antiviral mechanisms was determined using different concentrations of the *U. lactuca* extract (0.0312, 0.0156, 0.0078, and 0.0039 mg/mL) (Table 2). The tested *U. lactuca* concentration of 0.0312 mg/mL showed potential anti-SARS-CoV-2 activity that was primarily mediated by virucidal activity (64%), as well as interference with viral replication (33%) and viral adsorption (31%).

## 3. Discussion

The season of algae collection significantly affects the extraction yield, as algae collected in July gave a higher yield, as it was the active growth period of algae. Various methods can be applied to the *U. lactuca* extract for the active constituent that will be analyzed. In our study, we employed the hot water in the same way as Mhatre et al. [23]. The hot water, NaCO_3_, and NaOH methods for ulvan extraction were performed, and the researchers reported that the maximum yield was observed with the hot water extraction method [24,25]. Additionally, variations in concentration ratios, extraction temperature, and extraction time are important parameters to be studied [7]. Therefore, based on our results, the extraction conditions for the maximum extraction yield (~11.203%) were at 80 °C with a 1/20 algal powder/water ratio (g/mL) for 2 h. This finding is consistent with previous studies which have reported that the high *U. lactuca* extract yield with low degradation at temperatures of 80–90 °C and 1–3 h duration, and the yield percent is nearly similar to that of *Ulva ohnoi* (14.84%) [7,26].

Regarding the chemical composition of the aqueous extract from *U. lactuca*, carbon, hydrogen, and sulfur are the main elements with concentration percentages of 28.54%, 3.61%, and 7.08%, respectively. The main backbone of ulvan is carbon and hydrogen. At the same time, sulfur represents the sulfate content, a very important component within the cell walls of algae that plays a fundamental role in protecting plants under unfavorable environmental conditions, such as marine ecosystems. Additionally, an earlier study has shown a connection between *U. lactuca* sulfate concentration and its antioxidant activity and modulation of physiological stress [27,28]. However, the presence of nitrogen (2.04%) is due to the contact of *U. lactuca* with cell wall proteins [29].

The dried extract of *U. lactuca* contains unnecessary amounts of ash (44.004%); generally, the ash content in different *Ulva* species is reported to be relatively high due to its high sulfate content, as it was detected [30,31,32,33]. Sugars occur in 33.66%, higher than previously reported *Ulva lactuca* (27.41%) [34]. The increased photosynthetic activity of seaweeds during July, which enhances the growth rate and development, may be related to the high sugar content and reduced protein content (7.386%) [35,36]. The hydrophilic and hygroscopic nature of ulvan makes its water content relatively high (45.57%), as this percentage is compatible with its function in algae, where it acts as an osmoprotectant and forms a stiff gel to increase the stiffness of the cell wall while maintaining its flexibility [32]. Moreover, sulfate content was detected using the low-cost, fast, and environmentally-friendly turbidimetric method of Torres et al., 2021 [37]. The FT-IR spectrum is indistinguishable and similar to the IR spectra of sulfated polysaccharides extracted from different *Ulva* species [23,30,34,38,39,40,41,42]. The X-ray diffractogram showed sharp peaks at 2θ = 11°, 17°, 45°, 57°, and 72°, which indicated that ulvan has a semi-crystalline nature [32]. Ulvan has an indefinite backbone; hence, it has a highly branching structure. Therefore, ulvan has a disordered conformational structure due to its heterogeneous chemical composition, contributing to an amorphous region.

In contrast, the repeating aldobiouronic units could explain its crystalline region [34]. The EDX analysis confirmed the sulfated nature of the extracted polysaccharide. It also declared that the ulvan contains sulfuryl groups, as UV and FTIR analysis specified. Our results support the hypothesis that ulvan is a sulfated polysaccharide representing a unique component of the seaweed cell wall structure [32].

Regarding antiviral activity, the *U. lactuca* extract showed significant anti-SARS-CoV-2 activity which is related to the presence of sulfated polysaccharide ulvan. Our findings are consistent with previous studies on the polysaccharides extracted from seaweeds. Sulfated polysaccharides were the main topic of most research on the antiviral activity of marine algae [42,43,44,45,46,47]. Although intracellular activity has already been demonstrated for a sulfated polysaccharide, ulvan is a sulfated polysaccharide that carries significant negatively charged molecules, making it challenging for such compounds to have intracellular activity. Heteroglycuronan, the main component of ulvan, possesses antiviral properties against influenza A/PR/8/34 (H1N1) [44]. In the present study, aqueous extract from *U. lactuca* extracted from hot water exhibited potent antiviral activity mainly via interference with viral replication, consistent with observations from previous studies [45,46,48]. Furthermore, various human viruses, including the Hepatitis A virus (HAV-H_10_), the Coxsackie B4 virus, Herpes simplex virus types 1 and 2 (HSV-1), and measles virus, are susceptible to aqueous extracts of *U. lactate* [47,48,49,50]. Shefer et al. studied the anti-SARS-CoV-2 inhibition activities of crude ulvan extract in the Vero-E6 cells assay and reported that the extraction methods influence its chemical composition, cytotoxicity, and anti-SARS-CoV-2 activity [17].

Our findings suggest potential effects on viral replication, adsorption, and significant virucidal activity. According to several research studies, these negatively charged compounds disrupt the early stages of the proliferation cycle of the enveloped virus, thus preventing viral infection [45,51,52,53]. Other research indicated that sulfated polysaccharides could inhibit the synthesis of viral proteins [52] or different stages of the HSV life cycle [53]. The ulvan extract from *U. fasciata* exhibits good activity during the viral entry stage. Sepúlveda-Crespo et al. reported that the sulfate residues interfere with the positively charged viral glycoprotein domain and prevent the initial virus–cell interaction [54]. Chiu et al. found ulvan to prevent Japanese encephalitis virus (JEV) infection in the Vero cells by preventing virus adsorption and the virus from entering the cells [55]. In line with our work, Lopes et al. also demonstrated that certain sulfated polysaccharides from green seaweeds prevent virus replication [56].

## 4. Materials and Methods

### 4.1. Collection and Processing of Algal Samples

*Ulva lactuca*, which belongs to the *Ulvaceae* family, was chosen for this study as it is the most abundant *Ulva* species [30]. Dr. Fekry Ashour, a researcher at NIOF Egypt, collected it in July 2020 from the Gulf of Suez coast, Egypt, and identified it using the methods of [57,58]. Furthermore, the tested algae were evaluated via microscopic examination [59] and confirmed using the Algae Base website [60,61]. Following collection, all samples were washed several times with seawater to remove adhering debris, associated biota, and sand. After that, the samples were washed with tap water to remove salts. The algal samples were dried in the open air for 72 h in a shaded area, then dried in an oven (Memmert, Germany) at 105 °C for 3 h. The dried samples were ground into fine particles using a coffee grinder (Brown Mill, Berlin, Germany), sieved through 80-mesh screens, and stored in plastic bags at room temperature for further experiments.

### 4.2. Aqueous Extract from U. lactuca

The de-pigmentation of the collected seaweed powder was performed with 100 mL of hexane for 24 h with vigorous shaking at 3000 rpm before the seaweed powder was filtered. To remove soluble elements and unwanted impurities, the sample residue was immersed in 120 mL of 95% ethanol for 24 h at ambient temperature, with gentle stirring [62]. The residue was dried under vacuum at 60 °C for 3 h. Subsequently, the dried residual off-white weed was subjected to hot water extractions at temperatures of 60, 80, 90, and 100 °C at weed/water ratios (w g/v mL) of 1/10, 1/20, 1/40, and 1/50, respectively, for 30 min, 1 h, 2 h, and 3 h. After filtering through a cotton cloth, the aqueous extracts were centrifuged at 6708× *g* for 15 min in a cooling centrifuge (Centrikon T-124, Italy). The extracts were dialyzed for 48 h at 4 °C against distilled water to remove small elements. The aqueous extract was concentrated to 10%–20% of its initial concentration value using a rotary evaporator, followed by precipitation by adding four volumes of absolute ethanol to the concentrated aqueous extract at −20 °C. After 48 h, the precipitate was centrifuged in a cooling centrifuge for 15 min at 6708× *g*. Finally, the recovered residue was weighed to determine its fresh weight (gram fresh weight) to determine the optimum conditions for extraction. The extract was collected and dried in a vacuum oven at 60 °C. The dried *U. lactuca* extract was stored in sterilized falcon tubes for further experiments [27]. The extraction yield was determined for each condition using the equation:

*U. lactuca* extract yield (%) = (weight of extracted powder/weight of dry seaweed) × 100 to determine the best extraction conditions [22].

### 4.3. Analysis of the Extracted Ulvan

#### 4.3.1. Water Content

The water content related to the weight of the specimens was determined after 24 h of ignition at 103 °C in the oven. It was calculated as a percentage of the dry weight [63].

#### 4.3.2. Ash Content

Ash content was measured gravimetrically according to Lahaye and Jegou [29]. In a muffle furnace, 70 mg of the dried extract specimens were incinerated for 14 h at 550 °C.

#### 4.3.3. Elemental Analysis

By combusting the ulvan powder, the elements carbon, hydrogen, nitrogen, and sulfur were examined using an elemental analyzer (Vario MICRO Cube, Elementar, Germany).

#### 4.3.4. Protein Content

The soluble protein content was determined using the Lowry method [64]. In 2 mL of deionized water, 4 mg of the *U. lactuca* extract were dissolved. A 0.2 mL sample was added to 4 mL of Lowry’s solution. The mixture was allowed to stand for 10 min before adding 0.4 mL of a 50% Folin–Ciocalteu reagent, followed by incubation at ambient temperature for 30 min. The absorbance of mixtures was measured at 750 nm using a UV-VIS spectrophotometer (Jasco V 530, Japan). Based on a bovine serum albumin standard curve, the protein content was determined and represented as a percentage of ulvan dry weight.

#### 4.3.5. Total Sugar Content

A phenol–sulfuric acid assay was employed to assess the total sugar content in trifluoroacetic acid (TFA) hydrolysate [65]. The hydrolyzed sample of 0.5 mL was mixed with 2.5 mL of concentrated H_2_SO_4_ and 0.5 mL of a 5% (*v*/*v*) phenol solution. The mixture was stirred, boiled for 20 min, and then cooled to ambient temperature. An ultraviolet-visible (UV-VIS) spectrophotometer then measured the absorbance of the solution at 490 nm. The total sugar concentration was determined from a standard curve using various glucose concentrations. According to the glucose standard curve, total sugar was determined and represented as grams per 100 g of dry weight of the specimen.

#### 4.3.6. Sulfate Content

The sulfate content was determined using the turbidimetric method of Torres et al., 2021 [37], with the same accuracy as the traditional method [66]. Firstly, a barium chloride–gelatin reagent was prepared by adding 75 mg of gelatin powder and 25 mL of ultrapure water into a screw cap tube. Incubate at 80 °C for 10 min and vortex until complete homogenization, with the addition of 250 mg of BaCl_2_ to the gelatinous solution, and homogenize under stirring, then the reagent should be stored at 4 °C for further use, allowing the reagent to reach room temperature and become homogenized thoroughly before use.

In a 96-well clear polystyrene microplate, 20 μL of each of the following hydrolyzed ulvan solution, non-hydrolyzed ulvan solution, and 0.5 mol.L^−1^ HCl (negative control) was placed into each labeled well, which was already filled with 140 μL of 0.5 mol.L^−1^ HCl. After mixing, the absorbance of each well was determined at 405 nm in a microplate reader (Sunrise, TECAN, Inc., Woburn, MA, USA). An amount of 40 μL of the barium chloride–gelatin reagent was added. The contents of the wells were mixed. Then, incubation for the microplate for 20 min was performed, where we mixed the contents of the wells and measured the absorbance at 405 nm. These values correspond to the second reading. The organic sulfate (*OS*) content was calculated by correcting the absorbance values of each sample by subtracting the first reading from the second reading, using a standard curve of sulfate salt and the following equation.

$$OS=\left(µH-µN\right)\pm \sqrt{{\sigma H}^{2}+{\sigma N}^{2}}$$

where µ*H* and σ*H* are, respectively, the mean and standard deviation of the total sulfate percentages obtained with the hydrolyzed samples, and µ*N* and σ*N* are, respectively, the mean and standard deviation of the free sulfate percentages obtained with the non-hydrolyzed samples.

### 4.4. Characterization of the U. lactuca Extract

#### 4.4.1. Monosaccharide Composition Determination

The acid hydrolysis was performed on the samples following the modified [67]. An amount of 13 mg of the *U. lactuca* extract was transferred to a reaction tube with 5 mL of 4 M TFA. Hydrolyses were carried out for 6 h at 124 °C in the oven. At 40 °C, the hydrolysate was subsequently evaporated. HPLC (Agilent, California, USA) was used to determine the monosaccharide composition in the hydrolysate after it was dried. The apparatus is outfitted with a Binary HPLC pump with an injector, a refractive index detector (RI, 2410) at 35 °C, and a software monitor with the Breeze 2 HPLC program. Supelco supplied the LC-NH2 column (SUPELCOILTM LC- NH2, 250 4.6 mm, 5 μm). The column effluent was monitored using a refractive index detector. The mobile phase acetonitrile/water (85:15) solvent system performed at a 1.5 mL/min flow rate. Ten µL samples were injected into HPLC using HPLC grade water as the eluent at a flow rate of 1 mL min^−1^. Chromatographic peaks were recognized via comparison with reference sugars (galactose, fructose, rhamnose, and glucose) for monosaccharide determination. This goes in harmony with Madany et al. (2021), who recorded nearly the same monosaccharides throughout different seasons of the *Ulva* spp. collection [63]. Different concentrations of each standard were achieved via dilution with water, and the calibration curves were then constructed by graphing the peak area vs. the volume injected.

#### 4.4.2. FTIR

The functional groups in the aqueous extract from *U. lactuca* were detected using an FTIR spectrometer (Bruker, Alpha, Germany) configured with the attenuated total reflectance (ATR) method. The transmission spectra were identified using KBr pellets (Merck^®^, Rahway, NJ, USA) comprising 2.5 mg of the extract powder. After removing the atmospheric background interferences, the spectra were generated in the 4000–400 cm^−1^ range [68].

#### 4.4.3. XRD

Information regarding the crystalline characters of the *U. lactuca* extract was acquired using an X-ray diffractometer (Bruker D2 Phaser) with Cu Kα (λ = 1.5412 Å) radiation 2θ ranging from 5° to 85° at 40 kV and 30 mA.

#### 4.4.4. SEM and EDX

The *U. lactuca* extract was examined using high-resolution scanning electron microscopy (HRSEM; JSM-IT 200, Jeol, Japan) under a high vacuum and an acceleration voltage of 15 kV to understand more details about the morphological characteristics and the surface texture. The sample was coated with gold (15 Å) for 2 min via physical vapor deposition to be prepared for SEM analysis. The EDX spectrometer was employed to detect the weight and atomic percentages of the different elements in the specimen concerning the emitted X-rays [68].

### 4.5. Cytotoxicity Assay

The cytotoxic activity of the *U. lactuca* extract was tested in the Vero E6 cells using the MTT (3-[4,5-dimethylthiazol-2-yl]-2,5-diphenyl tetrazolium bromide) method with minor modifications. The tested viral strain was recovered from a nasal swab collected from a patient admitted to Almaza Military Hospital and was fully characterized (NCBI Virus GenBank Accession No. MT994983, Submitter Seadawy, M.G., et al.). A Biosafety Level-3 facility was used for viral culture procedures.

Briefly, the cells were seeded (3 × 10^5^ cells/mL) in 96-well plates, followed by 24 h of incubation at 37 °C in 5% CO_2_. The cells were then treated with different concentrations of extracted ulvan (0.0001 to 0.0009 g/mL) according to the concentrations’ range employed in previous studies [55,69,70]. Each experiment was performed in triplicate. The supernatant was removed after a further 24 h, and the cell monolayers were then washed three times with sterile PBS before being treated with the MTT solution, followed by incubation at 37 °C for 4 h before media aspiration. Each well received 200 µL of acidified isopropanol (0.04 M HCl in absolute isopropanol) to dissolve the formed formazan crystals. The absorbance of formazan solutions was determined at a maximum wavelength of 540 nm, using 620 nm as the standard wavelength. The percentage of cytotoxicity was determined from the following equation: By plotting % cytotoxicity versus sample concentration, we calculated the cytotoxic concentration 50 (CC_50_).

$$\%\;Cytotoxicity=\frac{\left(absorbance\;of\;cells\;without\;treatment-absorbance\;of\;cells\;with\;treatment\right)\times 100}{absorbance\;of\;cells\;without\;treatment}$$

### 4.6. Plaque Reduction Assay

The assay was performed according to Hayden et al. [71]. The SARS-CoV-2 virus was diluted to give approximately 10^6^ PFU/well, mixed with the harmless concentration of the *U. lactuca* extract at an approximate ten-fold dilution of CC_50_, followed by serial two-fold dilutions (0.0312, 0.0156, 0.0078, and 0.0039 mg/mL) and incubation for 1 h at 37 °C before being added to the cells. The growth medium was discarded from the plates, and the cells were inoculated with a 100 µL/well virus with the tested *U. lactuca*. The untreated cell culture served as a negative control group (the cells were inoculated with a 100 µL/well virus without the extract). After virus adsorption, 3 mL of DMEM plus 2% agarose and the tested extract were transferred to the cell monolayer. The plates were allowed to solidify before incubating at 37 °C for 3–4 days until viral plaques formed. The 10% formalin solution was added for fixation, and staining was performed with a 0.1% crystal violet solution. The control wells contained Vero E6 cells inoculated with the untreated virus. Finally, the plaque-forming unit was counted, and the percentage inhibition was calculated as follows:

$$\%\;inhibition=\frac{\left(Viral\;count\;untreated-viral\;count\;treated\right)\times 100}{Viral\;count\;untreated}$$

### 4.7. Mechanism of Antiviral Action

#### 4.7.1. Viral Replication

The virus was diluted to give 10^6^ PFU/well and applied directly to the cells, which were incubated for 1 h at 37 °C. The unadsorbed viral particles were removed by washing the cells three times with supplements for free-medium ulvan, starting with the concentration that resulted in >99.9% inhibition. After virus adsorption, 3 mL of DMEM plus 2% agarose and the tested extract in the same concentrations used in the plaque reduction assay were added to the cell monolayer. Plates were allowed to solidify before incubating at 37 °C for 3–4 days until viral plaques formed. The 10% formalin fixative solution was added, and subsequently, staining was conducted. The percentage inhibition was calculated as mentioned above.

#### 4.7.2. Viral Adsorption

The *U. lactuca* extract was applied at various concentrations in the 200 µL medium. The same concentrations were used as in the plaque reduction assay, beginning with the highest inhibitory value, and co-incubated with the cells for 2 h at 40 °C. After washing the cells three times with a supplement-free medium, the virus diluted to 10^6^ PFU/well was incubated with the pretreated cells for 1 h before adding 3 mL of DMEM supplemented with 2% agarose. Plates were processed as described above. The percentage inhibition was calculated as mentioned above.

#### 4.7.3. Virucidal Activity

A 200 µL serum-free DMEM volume containing 10^6^ PFU forming SARS-CoV-2 was added to the tested ulvan extract, resulting in high viral inhibition. Following 1 h of incubation, the mixture was diluted three times using the serum-free media, each time by a factor of 10, resulting in virtually no ulvan, but still allowing virus particles to proliferate on the VERO cells. The VERO cell monolayer was then treated with 100 L of each dilution. After 1 h of contact time, a DMEM overlayer was added to the cell monolayer. The plates were allowed to solidify before being placed in an incubator at 37 °C to promote the formation of viral plaques. They were fixed and stained as described above.

### 4.8. Statistical Analysis

Statistical analysis was performed using GraphPad Prism version 9.4.1 (San Diego, CA, USA). A one-way analysis of variance (ANOVA) was performed, followed by a Tukey-Kramer post hoc test to compare the different investigated groups. The *p*-value < 0.05 is statistically significant.

## 5. Conclusions

This is a pioneer study on the antiviral activity of an aqueous extract from *Ulva lactuca* against SARS-CoV-2. This study shows the effect of different conditions on the aqueous extraction of *U. lactuca* collected from the Gulf of Suez, Egypt. Based on our findings, the aqueous extract from *U. lactuca* has potential anti-SARS-CoV-2 activity by interfering with the viral attachment, adsorption, and replication processes. Therefore, *U. lactuca* could be a promising source of bioactive substances for preclinical study in the drug development process to control COVID-19.

## Figures and Tables

**Table 1:** Proximate chemical analysis and monosaccharide composition of the *Ulva lactuca* aqueous extract.

Parameter Measured	Value ± SE (%)
Yield	11.203 ± 0.32
Fresh w.t	6.175 ± 0.21
Water content	45.57 ± 0.292
Dry w.t	3.361 ± 0.06
**Chemical composition (%) Dry weight**	
Ash	44.004 ± 0.417
Total Sugars	33.66 ± 0.65
Protein	7.386 ± 0.41
Sulfate content	14.95 ± 0.24
**Element composition**	
C content	28.54
H content	3.61
N content	2.04
S content	7.08
**Sugar composition (mol%)**	
Rhamnose	32.88
Galactose	25.46
Fructose	28.25
Glucose	13.41

**Table 2:** The mechanisms of antiviral action of *U. lactuca* extract.

Mode of Action	*Ulva lactuca* Extract Conc. (mg/mL)	Viral Count Pre-Treatment (PFU/mL)	Viral Count Post-Treatment (PFU/mL)	Inhibition % *
**Viral Replication**	0.0312	6 × 10^5^	4 × 10^5^	33.3
	0.0156		4.3 × 10^5^	28.3
	0.0078		4.6 × 10^5^	23.3
	0.0039		5.5 × 10^5^	8.3
**Viral Adsorption**	0.0312	4.5 × 10^5^	3.1 × 10^5^	31.1
	0.0156		3.4 × 10^5^	24.4
	0.0078		3.7 × 10^5^	17.7
	0.0039		4.1 × 10^5^	8.8
**Virucidal**	0.0312	5 × 10^5^	1.8 × 10^5^	64
	0.0156		3.3 × 10^5^	34
	0.0078		3.9 × 10^5^	22
	0.0039		4.3 × 10^5^	14

* The experiments were performed in three independent replicates. Data were represented as mean ± SD. ANOVA test showed a significant difference among means (*p* ˂ 0.001).

## Data Availability

All data generated or analyzed during this study are included in this published article.

## References

[1] Domínguez H. (2013). Algae as a Source of Biologically Active Ingredients for the Formulation of Functional Foods and Nutraceuticals. Functional Ingredients from Algae for Foods and Nutraceuticals.

[2] Moreira A., Cruz S., Marques R., Cartaxana P. (2022). The Underexplored Potential of Green Macroalgae in Aquaculture. Rev. Aquacult..

[3] Gheda S., El-Sheekh M.M., Abou-Zeid A. (2018). In Vitro Anticancer Activity of Polysaccharide Extracted from the Red Alga *Jania rubens* Against Breast and Colon Cancer Cell Lines. Asian Pac. J. Trop. Med..

[4] El-Nabi S.H., Elhiti M., El-Sheekh M. (2020). A New Approach for COVID-19 Treatment by micro-RNA. Med. Hypotheses.

[5] Wei Q., Fu G., Wang K., Yang Q., Zhao J., Wang Y., Ji K., Song S. (2022). Advances in Research on Antiviral Activities of Sulfated Polysaccharides from Seaweeds. Pharmaceuticals.

[6] Li X., Gong Y., Yao W., Chen X., Xian J., You L., Fardim P. (2021). Structural Characterization and Protective Effects of Polysaccharide from *Gracilaria lemaneiformis* on LPS-Induced Injury in IEC-6 Cells. Food Chem. X.

[7] Huang L.X., Shen M.Y., Morris G.A., Xie J.H. (2019). Sulfated Polysaccharides: Immunomodulation and Signaling Mechanisms. Trends Food Sci. Technol..

[8] Wang W., Wang S.X., Guan H.S. (2012). The Antiviral Activities and Mechanisms of Marine Polysaccharides: An Overview. Mar. Drugs.

[9] Cindana Mo’o F.R., Wilar G., Devkota H.P., Wathoni N. (2020). Ulvan, a Polysaccharide from Macroalga *Ulva* sp.: A Review of Chemistry, Biological Activities and Potential for Food and Biomedical Applications. Appl. Sci..

[10] Yeo C., Kaushal S., Yeo D. (2020). Enteric Involvement of Coronaviruses: Is Faecal-Oral Transmission of SARS-CoV-2 Possible?. Lancet Gastroenterol. Hepatol..

[11] Seadawy M.G., Binsuwaidan R., Alotaibi B., El-Masry T.A., El-Harty B.E., Gad A.F., Elkhatib W.F., El-Bouseary M.M. (2022). The Mutational Landscape of SARS-CoV-2 Variants of Concern Recovered from Egyptian Patients in 2021. Front. Microbiol..

[12] Clausen T.M., Sandoval D.R., Spliid C.B., Pihl J., Perrett H.R., Painter C.D., Narayanan A., Majowicz S.A., Kwong E.M., McVicar R.N. (2020). SARS-CoV-2 Infection Depends on Cellular Heparan Sulfate and ACE2. Cell.

[13] Zhang L.P., Mann M., Syed Z.A., Reynolds H.M., Tian E., Samara N.L., Zeldin D.C., Tabak L.A., Hagen K.G.T. (2021). Furin Cleavage of the SARS-CoV-2 Spike Is Modulated by O-Glycosylation. Proc. Natl. Acad. Sci. USA.

[14] Xu X., Chen P., Wang J., Feng J., Zhou H., Li X., Zhong W., Hao P. (2020). Evolution of the Novel Coronavirus from the Ongoing Wuhan Outbreak and Modeling of Its Spike Protein for Risk of Human Transmission. Sci. China Life Sci..

[15] Balachandar V., Mahalaxmi I., Subramaniam M., Kaavya J., Senthil Kumar N., Laldinmawii G., Narayanasamy A., Reddy P.J.K., Sivaprakash P., Kanchana S. (2020). Follow-Up Studies in COVID-19 Recovered Patients—Is It Mandatory?. Sci. Total Environ..

[16] Mitrani R.D., Dabas N., Goldberger J.J. (2020). COVID-19 Cardiac Injury: Implications for Long-Term Surveillance and Outcomes in Survivors. Heart Rhythm.

[17] Shefer S., Robin A., Chemodanov A., Lebendiker M., Bostwick R., Rasmussen L., Lishner M., Gozin M., Golberg A. (2021). Fighting SARS-CoV-2 with Green Seaweed *Ulva* sp. Extract: Extraction Protocol Predetermines Crude Ulvan Extract Anti-SARS-CoV-2 Inhibition Properties in In Vitro Vero-E6 Cells Assay. PeerJ.

[18] Wu C., Liu Y., Yang Y., Zhang P., Zhong W., Wang Y., Wang Q., Xu Y., Li M., Li X. (2020). Analysis of therapeutic targets for SARS-CoV-2 and discovery of potential drugs by computational methods. Acta Pharm. Sin. B.

[19] Gudima G., Kofiadi I., Shilovskiy I., Kudlay D., Khaitov M. (2023). Antiviral Therapy of COVID-19. Int. J. Mol. Sci..

[20] Baum A., Fulton B.O., Wloga E., Copin R., Pascal K.E., Russo V., Giordano S., Lanza K., Negron N., Ni M. (2020). Antibody Cocktail to SARS-CoV-2 Spike Protein Prevents Rapid Mutational Escape Seen with Individual Antibodies. Science.

[21] Hilgenfeld R., Peiris M. (2013). From SARS to MERS: 10 Years of Research on Highly Pathogenic Human Coronaviruses. Antiviral Res..

[22] Yaich H., Amira A.B., Abbes F., Bouaziz M., Besbes S., Richel A., Blecker C., Attia H., Garna H. (2017). Effect of Extraction Procedures on Structural, Thermal and Antioxidant Properties of Ulvan from *Ulva Lactuca* Collected in Monastir Coast. Int. J. Biol. Macromol..

[23] Mhatre A., Navale M., Trivedi N., Pandit R., Lali A.M. (2018). Pilot Scale Flat Panel Photobioreactor System for Mass Production of *Ulva lactuca* (Chlorophyta). Bioresour. Technol..

[24] Robic A., Rondeau-Mouro C., Sassi J.-F., Lerat Y., Lahaye M. (2009). Structure and Interactions of Ulvan in the Cell Wall of the Marine Green Algae *Ulva rotundata* (Ulvales, Chlorophyceae). Carbohydr. Polym..

[25] Wahlström N., Nylander F., Malmhäll-Bah E., Sjövold K., Edlund U., Westman G., Albers E. (2020). Composition and Structure of Cell Wall Ulvans Recovered from *Ulva* Spp. along the Swedish West Coast. Carbohydr. Polym..

[26] Ponce M., Zuasti E., Anguís V., Fernández-Díaz C. (2020). Effects of the Sulfated Polysaccharide Ulvan from *Ulva ohnoi* on the Modulation of the Immune Response in Senegalese Sole (*Solea senegalensis*). Fish Shellfish Immunol..

[27] Peasura N., Laohakunjit N., Kerdchoechuen O., Wanlapa S. (2015). Characteristics and Antioxidant of *Ulva Intestinalis* Sulphated Polysaccharides Extracted with Different Solvents. Int. J. Biol. Macromol..

[28] Qi H., Sun Y. (2015). Antioxidant Activity of High Sulfate Content Derivative of Ulvan in Hyperlipidemic Rats. Int. J. Biol. Macromol..

[29] Lahaye M., Robic A. (2007). Structure and Functional Properties of Ulvan, a Polysaccharide from Green Seaweeds. Biomacromolecules.

[30] Ibrahim M.I.A., Amer M.S., Ibrahim H.A.H., Zaghloul E.H. (2022). Considerable Production of Ulvan from *Ulva lactuca* with Special Emphasis on Its Antimicrobial and Anti-fouling Properties. Appl. Biochem. Biotechnol..

[31] Ray B., Lahaye M. (1995). Cell-Wall Polysaccharides from the Marine Green Alga *Ulva Rigida* (Ulvales, Chlorophyta). Extraction and Chemical Composition. Carbohydr. Res..

[32] Alves A., Caridade S.G., Mano J.F., Sousa R.A., Reis R.L. (2010). Extraction and Physico-Chemical Characterization of a Versatile Biodegradable Polysaccharide Obtained from Green Algae. Carbohydr. Res..

[33] Costa C., Alves A., Pinto P.R., Sousa R.A., Borges da Silva E.A., Reis R.L., Rodrigues A.E. (2012). Characterization of Ulvan Extracts to Assess the Effect of Different Steps in the Extraction Procedure. Carbohydr. Polym..

[34] Olasehinde T.A., Mabinya L.V., Olaniran A.O., Okoh A.I. (2019). Chemical Characterization of Sulfated Polysaccharides from *Gracilaria gracilis* and *Ulva lactuca* and Their Radical Scavenging, Metal Chelating, and Cholinesterase Inhibitory Activities. Int. J. Food Prop..

[35] Fareeha A., Atika A., Aliya R. (2013). Protein Extraction from *Ulva lactuca* and *Padinapavonica* Found at Buleji Coast, Karachi. Pak. Int. J. Phycol. Phycochem..

[36] Rico J.M., Fernández C. (1996). Seasonal Nitrogen Metabolism in an Intertidal Population of *Gelidium latifolium* (Gelidiaceae, Rhodophyta). Eur. J. Phycol..

[37] Torres P.B., Nagai A., Jara C.E.P., Santos J.P., Chow F., Santos D.Y.A.C. (2021). Determination of sulfate in algal polysaccharide samples: A step-by-step protocol using microplate reader. Ocean. Coast. Res..

[38] Quemener B., Lahaye M., Bobin-Dubigeon C. (1997). Sugar Determination in Ulvans by a Chemical-Enzymatic Method Coupled to High-Performance Anion Exchange Chromatography. J. Appl. Phycol..

[39] Hung Y.R., Chen G.W., Pan C.L., Lin H.V. (2021). Production of Ulvan Oligosaccharides with Antioxidant and Angiotensin-Converting Enzyme-Inhibitory Activities by Microbial Enzymatic Hydrolysis. Fermentation.

[40] Figueira T.A., da Silva A.J.R., Enrich-Prast A., Yoneshigue-Valentin Y., de Oliveira V.P. (2020). Structural Characterization of Ulvan Polysaccharide from Cultivated and Collected *Ulva Fasciata* (Chlorophyta). Adv. Biosci. Biotechnol..

[41] Jagtap A.S., Parab A.S., Manohar C.S., Kadam N.S. (2022). Prebiotic Potential of Enzymatically Produced Ulvan Oligosaccharides Using Ulvan Lyase of *Bacillus subtilis*, NIOA181, a Macroalgae-Associated Bacteria. J. Appl. Microbiol..

[42] Tian H., Yin X., Zeng Q., Zhu L., Chen J. (2015). Isolation, Structure, and Surfactant Properties of Polysaccharides from *Ulva lactuca* L. from South China Sea. Int. J. Biol. Macromol..

[43] Mendes G.S., Soares A.R., Martins F.O., Albuquerque M.C., Costa S.S., Yoneshigue-Valentin Y., Gestinari L.M., Santos N., Romanos M.T. (2010). Antiviral Activity of the Green Marine Alga *Ulva fasciata* on the Replication of Human Metapneumovirus. Rev. Inst. Med. Trop. Sao Paulo.

[44] Jiao G., Yu G., Wang W., Zhao X., Zhang J., Ewart S.H. (2012). Properties of Polysaccharides in Several Seaweeds from Atlantic Canada and Their Potential Anti-influenza Viral Activities. J. Ocean Univ. China.

[45] Wang H., Ooi E.V., Ang P.O. (2008). Antiviral Activities of Extracts from Hong Kong Seaweeds. J. Zhejiang Univ. Sci. B.

[46] Damonte E., Neyts J., Pujol C.A., Snoeck R., Andrei G., Ikeda S., Witvrouw M., Reymen D., Haines H., Matulewicz M.C. (1994). Antiviral Activity of a Sulphated Polysaccharide from the Red Seaweed *Nothogenia fastigiata*. Biochem. Pharmacol..

[47] Soares A.R., Robaina M.C.S., Mendes G.S., Silva T.S.L., Gestinari L.M.S., Pamplona O.S., Yoneshigue-Valentin Y., Kaiser C.R., Romanos M.T.V. (2012). Antiviral Activity of Extracts from Brazilian Seaweeds Against Herpes Simplex Virus. Rev. Bras. Farmacogn..

[48] Zhu W., Ooi V.E.C., Chan P.K.S., Ang P.O. (2003). Isolation and Characterization of a Sulfated Polysaccharide from the Brown Alga *Sargassum patens* and Determination of Its Anti-herpes Activity. Biochem. Cell Biol..

[49] Shi Q., Wang A., Lu Z., Qin C., Hu J., Yin J. (2017). Overview on the Antiviral Activities and Mechanisms of Marine Polysaccharides from Seaweeds. Carbohydr. Res..

[50] Morán-Santibañez K., Cruz-Suárez L.E., Ricque-Marie D., Robledo D., Freile-Pelegrín Y., Peña-Hernández M.A., Rodríguez-Padilla C., Trejo-Avila L.M. (2016). Synergistic Effects of Sulfated Polysaccharides from Mexican Seaweeds Against Measles Virus. BioMed Res. Int..

[51] Witvrouw M., de Clercq E. (1997). Sulfated Polysaccharides Extracted from Sea Algae as Potential Antiviral Drugs. Gen. Pharmacol..

[52] González M.E., Alarcón B., Carrasco L. (1987). Polysaccharides as Antiviral Agents: Antiviral Activity of Carrageenan. Antimicrob. Agents Chemother..

[53] Huleihel M., Ishanu V., Tal J., Arad S. (2001). Antiviral Effect of Red Microalgal Polysaccharides on Herpes Simplex and Varicella Zoster Viruses. J. Appl. Phycol..

[54] Sepúlveda-Crespo D., Ceña-Díez R., Jiménez J.L., Ángeles Muñoz-Fernández M. (2017). Mechanistic Studies of Viral Entry: An Overview of Dendrimer-Based Microbicides as Entry Inhibitors Against Both HIV and HSV-2 Overlapped Infections. Med. Res. Rev..

[55] Chiu Y.H., Chan Y.L., Li T.L., Wu C.J. (2012). Inhibition of Japanese Encephalitis Virus Infection by the Sulfated Polysaccharide Extracts from *Ulva lactuca*. Mar. Biotechnol..

[56] Lopes N., Ray S., Espada S.F., Bomfim W.A., Ray B., Faccin-Galhardi L.C., Linhares R.E.C., Nozawa C. (2017). Green Seaweed Enteromorpha Compressa (Chlorophyta, Ulvaceae) Derived Sulphated Polysaccharides Inhibit Herpes Simplex Virus. Int. J. Biol. Macromol..

[57] Aleem A.A. (1978). Contributions to the Study of the Marine Algae of the Red Sea. I—The Algae in the Neighborhood of Al-Ghardaqa, Egypt (*Cyanophyceae*, *Chlorophyceae*, and *Phaeophyceae*). Bull. Fac. Sci. (Jeddah King Abdul Aziz Univ.).

[58] Aleem A.A. (1993). Marine Algae in Alexandria, Egypt.

[59] Lipkin Y.S. (2002). Marine Algae and Seagrasses of the Dahlak Archipelago, Southern Red Sea. Nova Hedwig..

[60] Braune W. (2008). Meeresalgen: Ein Farbbildführer zu den Verbreiteten Benthischen Grün-, Braun- und Rotalgen der Weltmeere [Seaweeds: A Colour Guide to the Widespread Benthic Green, Brown and Red Algae of the World’s Oceans].

[61] Guiry M.D., Guiry G.M. (2018). Algae Base. Worldwide Electronic Publication.

[62] Huang H.J., Ramaswamy S., Al-Dajani W.W., Tschirner U. (2010). Process Modeling and Analysis of Pulp Mill-Based Integrated Biorefinery with Hemicellulose Pre-extraction for Ethanol Production: A Comparative Study. Bioresour. Technol..

[63] Madany M.A., Abdel-Kareem M.S., Al-Oufy A.K., Haroun M., Sheweita S.A. (2021). The Biopolymer Ulvan from *Ulva fasciata*: Extraction towards Nanofibers Fabrication. Int. J. Biol. Macromol..

[64] Lowry O.H., Rosebrough N.J., Farr A.L., Randall R.J. (1951). Protein Measurement with the Folin Phenol Reagent. J. Biol. Chem..

[65] DuBois M., Gilles K.A., Hamilton J.K., Rebers P.A., Smith F. (1956). Colorimetric Method for Determination of Sugars and Related Substances. Anal. Chem..

[66] Dodgson K.S., Price R.G. (1962). A note on the determination of the ester sulfate content of sulfated polysaccharides. Biochem. J..

[67] Toskas G., Heinemann S., Heinemann C., Cherif C., Hund R.D., Roussis V., Hanke T. (2012). Ulvan and Ulvan/Chitosan Polyelectrolyte Nanofibrous Membranes as a Potential Substrate Material for the Cultivation of Osteoblasts. Carbohydr. Polym..

[68] Saleh Amer M., Zaghloul E.H., Ibrahim M.I.A. (2020). Characterization of Exopolysaccharide Produced from Marine-Derived *Aspergillus terreus* SEI with Prominent Biological Activities. Egypt. J. Aquat. Res..

[69] Aguilar-Briseño J.A., Cruz-Suarez L.E., Sassi J.-F., Ricque-Marie D., Zapata-Benavides P., Mendoza-Gamboa E., Rodríguez-Padilla C., Trejo-Avila L.M. (2015). Sulphated Polysaccharides from *Ulva clathrata* and *Cladosiphon okamuranus* Seaweeds both Inhibit Viral Attachment/Entry and Cell-Cell Fusion, in NDV Infection. Mar. Drugs.

[70] Hardouin K., Bedoux G., Burlot A.S., Donnay-Moreno C., Bergé J.P., Nyvall-Collén P., Bourgougnon N. (2016). Enzyme-assisted extraction (EAE) for the production of antiviral and antioxidant extracts from the green seaweed *Ulva armoricana* (Ulvales, Ulvophyceae). Algal Res..

[71] Hayden F.G., Cote K.M., Douglas R.G. (1980). Plaque Inhibition Assay for Drug Susceptibility Testing of Influenza Viruses. Antimicrob. Agents Chemother..

